# Effect of Risankizumab Induction and Maintenance Therapy on the Rate of Hospitalization in Patients with Crohn’s Disease

**DOI:** 10.1016/j.gastha.2024.100603

**Published:** 2024-12-24

**Authors:** Brian G. Feagan, Remo Panaccione, Stefan Schreiber, Edward V. Loftus, Laurent Peyrin-Biroulet, Takehiro Arai, Wan-Ju Lee, Jenny Griffith, Jasmina Kalabic, Kristina Kligys, Si Xuan, Xiaomei Liao, Marc Ferrante

**Affiliations:** 1 Department of Gastroenterology, Western University, London, Ontario, Canada; 2 Alimentiv Inc, London, Ontario, Canada; 3 Department of Gastroenterology and Hepatology, University of Calgary, Calgary, Alberta, Canada; 4 Clinic for Internal Medicine, University Hospital Schleswig-Holstein, Kiel, Germany; 5 Division of Gastroenterology and Hepatology, Mayo Clinic College of Medicine and Science, Rochester, Minnesota; 6 Department of Gastroenterology, University of Lorraine, Inserm, NGERE, Nancy, France; 7 Groupe Hospitalier privé Ambroise Paré - Hartmann, Paris IBD center, Seine, France; 8 Gastroenterology Division, Toukatsu Tsujinaka Hospital, Abiko, Japan; 9 AbbVie Inc., North Chicago, Illinois; 10 Department of Gastroenterology & Hepatology, University Hospitals Leuven, KU Leuven, Leuven, Belgium

**Keywords:** Crohn’s Disease, Risankizumab, Interleukin-23 Inhibitor, Hospitalizations

## Abstract

**Background and Aims:**

In phase 3 induction (ADVANCE and MOTIVATE) and maintenance (FORTIFY) trials, risankizumab was shown to benefit symptom and endoscopic defined outcomes in patients with Crohn’s disease (CD). We examined the effects of risankizumab on the incidence of CD-related hospitalizations in these studies.

**Methods:**

Patients with active CD were randomized to intravenous (IV) risankizumab 600 or 1200 mg, or placebo at weeks 0, 4, and 8 in the 12-week induction studies. Clinical responders were rerandomized to maintenance with subcutaneous (SC) risankizumab 180 or 360 mg or placebo every 8 weeks for 52 weeks. Incidence of CD-related hospitalizations was compared between groups, expressed as proportions with an event during induction and event/100 person-years (PYs) during maintenance. An integrated analysis incorporated exposure time and occurrence of CD-related hospitalizations in induction and maintenance periods for labeled doses.

**Results:**

The incidence of CD-related hospitalizations was lower (3.2% or 1.9% vs 11.6%; *P* < .01) in the risankizumab IV 600- and 1200-mg groups vs placebo IV during induction. Through 52 weeks, the incidence rate per 100 PYs of CD-related hospitalizations was similar among treatment groups, with few events reported (n = 5–9 per group). In the integrated analysis, a lower incidence rate per 100 PYs of CD-related hospitalizations was observed in the risankizumab-treated groups (600 mg IV/360 mg SC: 9.6; 600 mg IV/180 mg SC: 7.9) vs placebo (40.0, *P* < .001).

**Conclusion:**

Risankizumab treatment resulted in reduced rates of CD-related hospitalization with treatment effect observed within 12 weeks of randomization.

## Introduction

Crohn’s disease (CD) is a chronic and progressive inflammatory disorder frequently resulting in disease-related complications, such as strictures, fistulas, and abscesses.^1^, ^2^, ^3^ Consequently, patients often require hospitalizations^4^ or surgery, with between 25%–55% and 18%–59% of patients experiencing at least one of these events within 5 years of diagnosis.^5^, ^6^, ^7^ Increased rates of hospitalizations and surgery account for substantial costs to patients, the health-care system, and society.^8^, ^9^, ^10^, ^11^, ^12^, ^13^, ^14^, ^15^, ^16^ For example, direct total costs to patients and society were estimated at $6.3 billion annually in the United States, with hospitalizations representing a substantial portion of these costs.^9^ In a pan-European study, costs were estimated at €6,768,173 over 5 years, with hospitalizations accounting for 50% of costs during the first year.^10^ In recent years, primary drivers of costs related to CD management have evolved from inpatient to outpatient care and advanced therapies.^8^^,^^14^ While the economic burden of CD has shifted over time, it remains substantial and inpatient care remains an important cost driver.^10^^,^^13^, ^14^, ^15^, ^16^, ^17^, ^18^

As CD presentation and management is highly variable, costs also differ by disease severity, clinical course, and treatment.^8^^,^^18^^,^^19^ For example, patients in remission usually have lower costs than those with more severe disease or who are refractory to treatment.^8^^,^^20^ Accordingly, an economic imperative exists to identify more effective management that reduces the burden of CD-related complications, hospitalizations, and surgeries. Approaches to improve disease management have taken 2 directions.

First, there has been considerable interest in the concept of treating to specific therapeutic targets using highly defined algorithms.^21^ Recently, recommended targets for CD include early control of symptoms followed by intermediate targets of symptom/biomarker-defined remission, with the ultimate long-term goal of endoscopic healing.^21^ Previous studies have demonstrated that endoscopic healing is associated with long-term clinical remission and decreased rates of CD-related surgeries.^22^, ^23^, ^24^ The second relevant approach is incorporating novel therapies into these algorithms. Despite availability of multiple advanced treatment options, many patients do not respond to or lose response to therapy over time, or experience side effects.^25^, ^26^, ^27^, ^28^ Studies of existing agents have shown inconsistent benefit for reduction of surgery and hospitalization, underscoring the need for better medical therapy and inclusion of CD-related hospitalizations as a key endpoint for clinical trials.^4^^,^^29^, ^30^, ^31^, ^32^

Risankizumab, a humanized immunoglobulin G1 monoclonal antibody specifically targeting interleukin-23 by binding the p19 subunit, was recently approved in the United States and the European Union for treatment of CD. Results from phase 3 induction (ADVANCE [NCT03105128] and MOTIVATE [NCT03104413]) and maintenance (FORTIFY [NCT03105102]) trials demonstrate that risankizumab induction therapy reduces the symptoms and endoscopic inflammation of CD, and these effects persist through 52 weeks of maintenance therapy.^33^^,^^34^ Given these benefits, we evaluated hospitalization rates in patients assigned to risankizumab induction and maintenance treatment or placebo in these phase 3 trials.

## Materials and Methods

### Study Design and Patients

Study designs, patient selection, and primary results from multinational ADVANCE, MOTIVATE, and FORTIFY clinical trials were previously reported.^33^^,^^34^ Specific information regarding eligibility criteria and treatment schedules can be found in Supplementary materials: Appendix A. Briefly, patients were randomized 2:2:1 in ADVANCE and 1:1:1 in MOTIVATE to receive intravenous (IV) risankizumab 600 mg, 1200 mg, or placebo. Responders to IV risankizumab were rerandomized 1:1:1 to receive risankizumab 360 mg SC, 180 mg SC, or placebo SC (withdrawal from IV risankizumab) every 8 weeks for 52 weeks, from week 12 of induction (eg, week 0 of maintenance) onward.

#### Analysis Study Population

Induction study analyses were conducted in the intention-to-treat 1A population defined as all randomized patients with eligible baseline Simple Endoscopic Scores for CD scores who received at least 1 dose of study drug during the 12-week induction period. For the maintenance study, the intention-to-treat 1A population analyzed at week 52 included rerandomized patients who achieved clinical response after receiving IV risankizumab for 12 weeks in ADVANCE or MOTIVATE and received at least 1 dose of SC study drug in FORTIFY.

### Outcomes

Occurrences of any CD-related hospitalizations were recorded during the clinical trials and are presented here as proportions of patients with an event during induction and as events per 100 person-years during maintenance. Hospitalization was defined as a hospitalization event for any reason or cause that was at least a 24-hour stay. Hospitalizations were recorded during the clinical trials by investigators, and the assessment of whether hospitalization events were related to CD was recorded by the investigators at their discretion and individual interpretation.

### Statistical Analysis

Baseline demographics and disease characteristics were summarized by treatment group. Number and percentage of patients and means and standard deviations were used to summarize categorical and continuous variables, respectively.

More detailed information regarding statistical analysis is available in Appendix B of the Supplementary Materials. Occurrence of CD-related hospitalizations after 12 weeks of induction treatment was assessed under overall type I error control at a significance level of α = 0.05 (2-sided) and were reported as the number and proportion of patients with at least 1 CD-related hospitalization; 95% confidence intervals for incidence rate were based on normal approximation to binomial distribution. *P* values were based on a Chi-square test comparing proportions of patients with hospitalizations through 12 weeks between each risankizumab treatment group (ie, IV 600 mg or IV 1200 mg) and IV placebo. Incidence rates for hospitalization during the maintenance period, among patients who were responders to risankizumab induction therapy, were calculated as number of patients with CD-related hospitalization events divided by time at risk (person-years [PYs]) and are reported as incident rate per 100 PYs. Incidence rate difference, 95% confidence intervals, and nominal *P* value were calculated to evaluate statistical significance of the difference between each risankizumab SC groups and treatment withdrawal placebo SC group. Analysis was based on as-observed data before receiving risankizumab rescue therapy.

An integrated analysis was conducted to understand the effects of the complete risankizumab treatment regimen on reductions in hospitalization events by incorporating exposure time and occurrence of CD-related hospitalization events in both induction and maintenance periods for labeled doses. A sensitivity analysis was also conducted by accounting for exposure time and event for patients who were clinical responders to induction placebo IV and received placebo SC in the maintenance period.

All analyses were performed using SAS Version 9.4 or later (SAS Institute Inc., Cary, NC). All authors had access to the study data and reviewed and approved the final manuscript.

### Ethical Requirements

All 3 trials were conducted in compliance with the protocol and according to International Conference on Harmonisation guidelines and the principles of the Declaration of Helsinki. Patients provided written informed consent. Trial protocols and informed consent were approved by independent ethics committees and institutional review boards at all study sites. All patients provided informed consent before study participation.

## Results

### Study Population

A total of 850 (placebo: 175; risankizumab 600 mg IV: 336; risankizumab 1200 mg IV: 339) and 569 (placebo: 187; risankizumab 600 mg IV: 191; risankizumab 1200 mg IV: 191) patients participated in the ADVANCE and MOTIVATE induction trials, respectively. Induction baseline characteristics were similar among treatment groups (Table), including baseline corticosteroid use which ranged from 29% to 36%. Approximately 58% of patients in ADVANCE had intolerance and/or inadequate response to at least 1 biologic therapy, while all enrolled patients in MOTIVATE had intolerance and/or inadequate response to biologic therapy.

**Table:** Baseline Characteristics.^33^^,^^a^

Characteristic	ADVANCE			MOTIVATE		
	PBO IV N = 175	RZB 600 mg IV N = 336	RZB 1200 mg IV N = 339	PBO IV N = 187	RZB 600 mg IV N = 191	RZB 1200 mg IV N = 191
Age (y), mean ± SD	37.1 ± 13.4	38.3 ± 13.3	37.0 ± 13.2	39.3 ± 13.5	40.2 ± 13.6	39.3 ± 12.9
Males, n (%)	88 (50.3)	189 (56.3)	183 (54.0)	99 (52.9)	92 (48.2)	102 (53.4)
Race						
White, n (%)	134 (76.6)	258 (76.8)	247 (72.9)	162 (86.6)	176 (92.1)	168 (88.0)
Asian, n (%)	31 (17.7)	65 (19.3)	74 (21.8)	15 (8.0)	8 (4.2)	14 (7.3)
Black or African American, n (%)	9 (5.1)	9 (2.7)	13 (3.8)	7 (3.7)	7 (3.7)	8 (4.2)
Native Hawaiian or other Pacific Islander	1 (0.6)	0	1 (0.3)	2 (1.1)	0	0
Multiple	0	4 (1.2)	4 (1.2)	1 (0.5)	0	1 (0.5)
CD duration (y), mean ± SD	8.2 ± 7.1	9.0 ± 8.8	8.9 ± 8.4	12.5 ± 9.7	10.9 ± 7.7	11.8 ± 9.0
CD location, n (%)						
Ileal only	19 (10.9)	52 (15.5)	54 (15.9)	26 (13.9)	33 (17.3)	21 (11.0)
Colonic only	70 (40.0)	115 (34.2)	118 (34.8)	73 (39.0)	75 (39.3)	74 (38.7)
Ileocolonic	86 (49.1)	169 (50.3)	167 (49.3)	88 (47.1)	83 (43.5)	96 (50.3)
Baseline CDAI, mean ± SD	319.2 ± 59.4	311.2 ± 62.4	311.5 ± 68.4	319.6 ± 69.8	310.7 ± 63.6	312.5 ± 61.2
Baseline corticosteroid use, n (%)	50 (28.6)	102 (30.4)	101 (29.8)	68 (36.4)	65 (34.0)	62 (32.5)
Failed >1 prior biologics, n (%)	56 (32.0)	95 (28.3)	101 (29.8)	99 (52.9)	99 (51.8)	103 (53.9)

CDAI, Crohn’s disease activity index; IV, intravenous; PBO, placebo; RZB, risankizumab; SD, standard deviation.

a Some data points presented in D'Haens G, et al.

Among patients enrolled in ADVANCE and MOTIVATE, 462 were clinical responders to 12-week induction therapy with risankizumab IV and entered the FORTIFY maintenance study; of these, 164 were rerandomized to withdrawal placebo SC, 157 to risankizumab 180 mg SC, and 141 to risankizumab 360 mg SC.

### Incidence of CD-Related Hospitalizations during the 12-Week Induction Period

**Figure 1:**
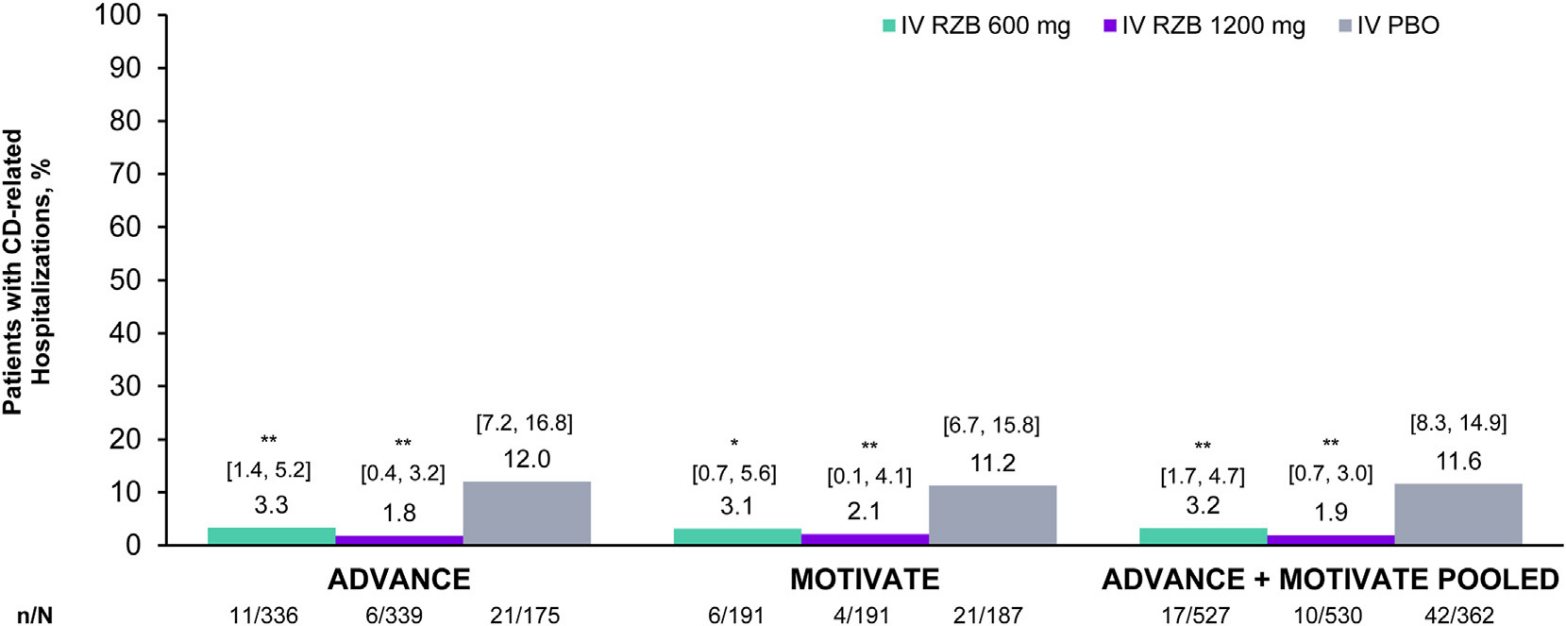
Proportion of patients with CD-related hospitalization during RZB induction. ∗*P* < .01, ∗∗*P* < .001. Pooled analysis included randomized eligible patients who had received at least 1 dose of study drug in 12-week induction from ADVANCE or MOTIVATE. 95% CIs based on the normal approximation to the binomial distribution. IV, intravenous; PBO, placebo; RZB, risankizumab.

Proportions of patients with CD-related hospitalizations were significantly lower in risankizumab IV arms compared to placebo IV in ADVANCE and MOTIVATE, respectively. In the pooled analysis, proportion of CD-related hospitalizations through week 12 was lower among patients in the risankizumab 600 mg IV group (3.2%) and risankizumab 1200 mg IV group (1.9%) vs patients in the placebo IV group (11.6%, *P* < .001) (Figure 1).

The proportion of patients who had a surgery event during the hospitalization was 35.3% in the risankizumab 600 mg IV group, 20.0% in the risankizumab 1200 mg IV group, and 21.4% in the placebo IV group (Table A1).

### Incidence of CD-Related Hospitalizations through the 52-Week Maintenance Period

**Figure 2:**
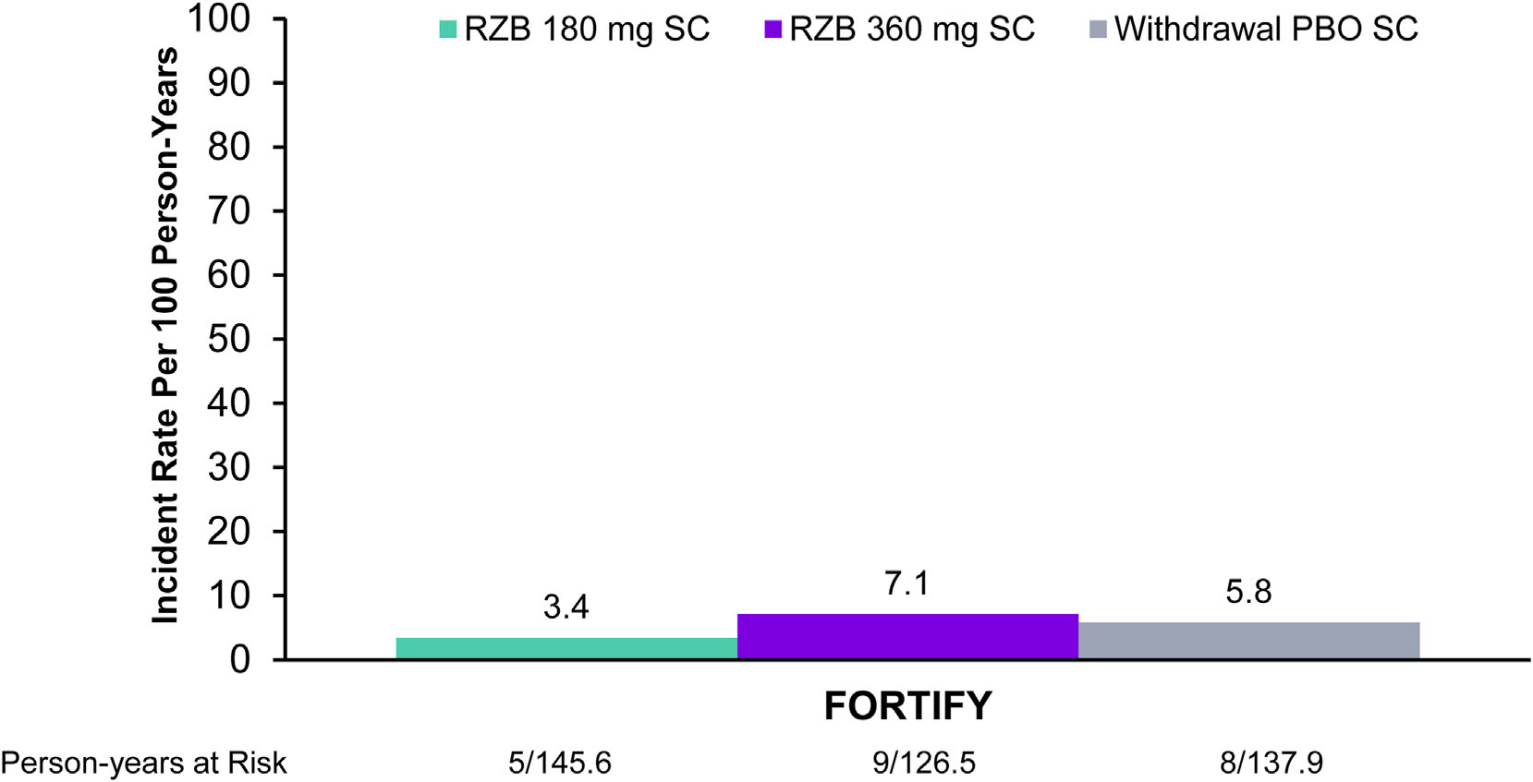
Exposure-adjusted occurrence of CD-related hospitalizations from week 0 through week 52 of the RZB maintenance period. Data presented as rate of occurrence per 100 person-years; rate was calculated as the n number of patients with a CD-related hospitalization event divided by the time at risk in person-years. PBO, placebo; RZB, risankizumab; SC, subcutaneous.

Incidence rates per 100 PYs of CD-related hospitalizations through 52 weeks were similar between risankizumab 180 and 360 mg SC and treatment withdrawal placebo SC groups, with very few CD-related hospitalizations reported (Figure 2). Number of patients with disease-related hospitalizations was 5, 9, and 8 in the risankizumab 180 and 360 mg SC arms and the withdrawal placebo SC arm, respectively.

The proportion of patients who had a surgery event during the maintenance hospitalization was 22.2% in the risankizumab 360 mg SC arm and 50.0% in the placebo SC arm. There were no surgery events during the maintenance hospitalization in the risankizumab 180 mg SC arm (Table A1).

### Integrated Analysis of CD-Related Hospitalizations Combining Both Induction and Maintenance Periods

**Figure 3:**
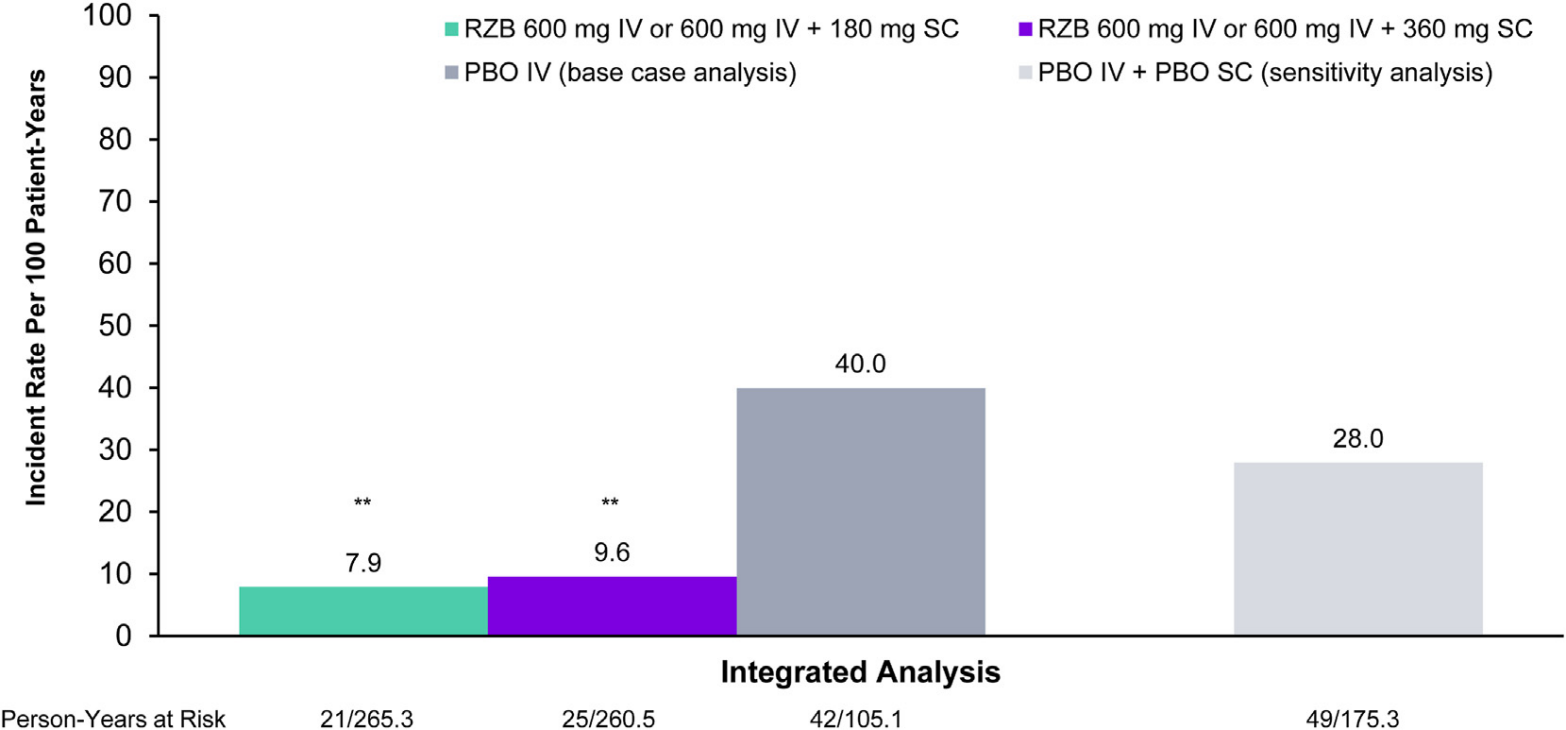
Exposure-adjusted occurrence of CD-related hospitalizations from an integrated analysis^a^ of all MOTIVATE, ADVANCE, and FORTIFY studies. ∗∗*P* < .001 for both PBO IV and PBO IV + PBO SC comparisons.^a^Patients with labeled dose (RZB 600 mg IV ± 180 mg SC or 360 mg SC) or placebo were followed from induction baseline in MOTIVATE and ADVANCE to end of maintenance period in FORTIFY. Time at risk was defined as number of person-years from date of the first dose of study drug to (1) first hospitalization event before patients received any RZB rescue therapy; (2) change in treatment of RZB not consistent with labeled dose (eg, from RZB 600 mg IV to RZB 1200 mg IV); (3) not achieving clinical response at week 12 or week 24 in MOTIVATE or ADVANCE with labeled dose of RZB; (4) change in treatment from placebo to RZB 1200 mg IV in MOTIVATE or ADVANCE; (5) entrance to the open-label extension study; (6) date of RZB rescue visit if receiving rescue therapy; (7) last available data if prematurely discontinued; (8) last dose + 140 days, whichever occurs earlier. IV, intravenous; PBO, placebo; RZB, risankizumab; SC, subcutaneous.

CD-related hospitalizations were observed at significantly lower rates in the risankizumab treatment groups (600 mg IV + 180 mg SC [7.9/100] and 600 mg IV + 360 mg SC [9.6/100] PYs) compared to placebo group (placebo IV only: 40/100 PYs) in the integrated analysis (Figure 3). Furthermore, similar results of lower rates of CD-related hospitalizations per 100 PYs in the risankizumab treatment groups vs placebo were observed in the sensitivity analysis (600 mg IV + 180 mg SC: 7.9 and 600 mg IV + 360 mg SC: 9.6 vs placebo IV + placebo SC: 28.0, both *P* < .001), which included patients who were responders to induction placebo IV and received placebo SC in the maintenance period in the comparator group (Figure 3).

## Discussion

Hospitalization and surgery remain relatively common among patients with CD despite improvements in treatment. As hospitalizations may be disruptive to patients’ lives, they are not a desired outcome from the patient perspective. Importantly, in addition to their negative effect on patient well-being, they substantially contribute to economic disease burden.^11^^,^^29^^,^^30^ The present study evaluated the effect of risankizumab induction and maintenance therapy on CD-related hospitalization rates. Here, the rate of disease-related hospitalizations was reduced early during treatment with significant reductions in hospitalization proportion observed among patients treated with IV risankizumab compared to IV placebo. These low hospitalization-event rates from the risankizumab arms were maintained through the 52-week observation period of the maintenance trial. Furthermore, in an integrated analysis incorporating induction and maintenance periods, a significantly lower CD-related hospitalization rate was observed in the active treatment arms relative to placebo. These findings add to previously published data on efficacy of risankizumab demonstrating consistent improvement relative to placebo in signs and symptoms of CD, mucosal inflammation, and quality of life.^33^, ^34^, ^35^

CD-related hospitalizations during the maintenance period were similar between risankizumab SC and treatment withdrawal placebo SC groups. However, the small number of hospitalization events that occurred overall during the 52-week maintenance period may have limited our ability to detect differences between groups. This may have been a consequence of the rerandomization design that specified continued exposure to drug in the active group and withdrawal of therapy to placebo in responders based on symptomatic improvement criteria.^34^ Notably, patients rerandomized to the treatment withdrawal placebo SC group had residual risankizumab exposure from IV induction therapy, as previously described.^34^ Furthermore, duration of risankizumab’s pharmacodynamic effects may exceed duration of pharmacokinetic effects.^34^ Thus, it is possible the interleukin-23 pathway was suppressed to a meaningful extent during the 52-week maintenance period among patients in the treatment withdrawal placebo SC group.

An additional consideration is that more disease worsening occurred in the placebo group as defined by the increased number of treatment-emergent adverse events of CD in the placebo SC (32 of 184, 17%) vs risankizumab SC treatment groups (19 of 179, 11% for 180 mg and 21 of 179, 12% for 360 mg).^34^ Withdrawal of these high-risk patients from the analysis population may have reduced our ability to detect differences in hospitalization rates between treatment groups.^34^ Availability of risankizumab rescue treatment may have prevented higher CD-related hospitalization rates in the maintenance study because rescue therapy may have been administered before patients experiencing worsening of CD severe enough to warrant hospitalization. If patients were hospitalized after receiving rescue therapy, those events would not have been included in these analyses. Therefore, comparable rates of CD-related hospitalizations between all treatment groups at week 52 may partially be explained by the potentially prolonged effect of risankizumab and availability of rescue therapy in the withdrawal placebo SC group.

Furthermore, an integrated analysis considering the patient flow through both induction and maintenance periods of treatment was further conducted to provide a complete picture of treatment impact on CD-related hospitalization rates. In the base case integrated analysis, observed CD-related hospitalization rate was significantly lower for risankizumab groups compared to placebo IV. The base case compared outcomes between risankizumab groups (ie, risankizumab 600 mg IV + 180 mg or 360 mg SC) and the placebo IV induction only, and patients who switched from placebo IV to risankizumab maintenance treatment were censored to avoid result interpretation bias. The base case analysis provides comparison to the background estimates of CD hospitalization rates in the most straightforward approach as patients in the comparator group received placebo IV only without further rerandomization. However, analyses utilizing only placebo IV induction data may underestimate the true rate of hospitalizations in the maintenance period, as the rate may be expected to increase if these patients with moderate-to-severe CD continue to lack receipt of active treatment. Therefore, a sensitivity analysis was subsequently conducted in which patients who responded to placebo IV induction treatment and continued to receive placebo SC in the maintenance period were included. Rate of hospitalizations in this subgroup of patients was lower than the rate observed among patients in the placebo IV induction-only group, but was still significantly higher compared to patients who received active risankizumab induction and maintenance treatment. Notably, sensitivity results may further underestimate CD hospitalization rate in patients receiving placebo as this patient population included placebo responders who may not be representative of the true placebo group. Additionally, during the induction period, patients were able to receive concomitant corticosteroids, including in the placebo group, at a stable dose; however, the distribution was similar across groups and is not expected to have impacted the results. Overall, both the integrated analysis results and primary analyses suggest patients who respond to treatment and achieve reduced rates of disease-related hospitalizations early during treatment may maintain reduced rates through 1 year of maintenance if active treatment is continued.

The effect of risankizumab on the reduced rate of CD-related hospitalization also implies a positive economic benefit. Cost of CD-related hospitalizations varies by region, ranging from an average of $2695 per event in Europe and $30,127 per event based on US data from 2022, with adjustments for inflation.^16^^,^^36^ In our study, the incidence rate of hospitalization was 9.6 of 100 PYs for patients treated with risankizumab 600 mg IV and 360 mg SC vs 40 of 100 PYs in placebo, based on the integrated analysis of both induction and maintenance data. A reduction in CD-related hospitalizations events of 30.4 of 100 PYs would result in a cost saving of $915,852 for every 100 patients treated with risankizumab for a year in the United States and $81,933 for every 100 patients treated with risankizumab for a year in Europe. Other factors should also be considered in understanding the full economic benefits, such as costs of treatment, indirect costs, and cost of hospitalization by country.

## Conclusion

There are strengths and limitations to this study. Strengths include the randomized clinical trial design with a large study sample size to minimize selection bias and longitudinal follow-up over 52 weeks. One limitation includes the small number of hospitalization events observed by week 52 as the study was not powered to evaluate this. Other limitations were the potential confounding effect of drug exposure in the withdrawal population that continued into the maintenance phase and the unknown outcome on patients who were lost to follow-up following withdrawal for disease worsening (ie, patients who were censored after receiving rescue therapy). Investigators determined whether a hospitalization was related to CD, and this could have varied between investigators, which may have impacted the number of events determined as CD-related. The number of surgical events occurring during a hospitalization was determined; however, whether the hospital admission was specifically for the surgical event could not be determined. Whether a hospitalization was classified as mild, moderate, or severe could not be determined. These results may not be generalizable to the wider population of patients with CD who were not enrolled in a clinical trial. Lastly, the number of observed surgery events was too small for statistical analysis and inclusion in this analysis but warrants future analyses to understand the full benefit of treatment with risankizumab on CD-related complications in clinical trial and real-world settings.

Risankizumab induction therapy significantly reduced CD-related hospitalizations as early as week 12 and continued to maintain low rates through week 52 in patients with active CD.

## Supplementary materials

Supplement_D1
