# Supplementary material for: Effect of Risankizumab Induction and Maintenance Therapy on the Rate of Hospitalization in Patients with Crohn’s Disease
Source: Gastro Hep Adv. 2024 Dec 24;4(4):100603. doi: 10.1016/j.gastha.2024.100603 (PMC11968275; doi:10.1016/j.gastha.2024.100603)
Supplement: Supplement_D1 [file mmc1.docx]

**Effect of Risankizumab Induction and Maintenance Therapy on the Rate of Hospitalization in Patients With Crohn’s Disease**

**Authors:** Brian G. Feagan,^1,2^ Remo Panaccione,^3^ Stefan Schreiber,^4^ Edward V. Loftus, Jr. ,^5^ Laurent Peyrin-Biroulet,^6^ Takehiro Arai,^7^ Wan-Ju Lee,^8^ Jenny Griffith,^8^ Jasmina Kalabic,^8^ Kristina Kligys,^8^ Si Xuan,^8^ Xiaomei Liao,^8*^ Marc Ferrante^9^

**Affiliations:** ^1^Western University, London, ON, Canada; ^2^Alimentiv Inc, London, ON, Canada; ^3^Department of Gastroenterology and Hepatology, University of Calgary, Calgary, Alberta, Canada; ^4^University Hospital Schleswig-Holstein, Kiel, Germany; ^5^Division of Gastroenterology and Hepatology, Mayo Clinic College of Medicine and Science, Rochester, Minnesota, USA; ^6^University of Lorraine, Inserm, NGERE, F-54000 Nancy, France; Groupe Hospitalier privé Ambroise Paré – Hartmann, Paris IBD center, 92200 Neuilly sur Seine, France; ^7^Toukatsu Tsujinaka Hospital, Abiko, Japan; ^8^AbbVie Inc., North Chicago, Illinois, USA; ^9^Department of Gastroenterology & Hepatology, University Hospitals Leuven, KU Leuven, Leuven, Belgium

*Former employee of AbbVie and current employee of Sanofi, Bridgewater, New Jersey, USA

**Funding:** Financial support for the study was provided by AbbVie. AbbVie participated in the interpretation of data, review, and approval of the manuscript. All authors contributed to the development of the manuscript and maintained control over the final content. No honoraria or payments were made for authorship.

**Supplemental Table 1. Summary of medical and surgical status of the first CD-related hospitalization during the 12-week induction and 52-week maintenance periods**

| **Treatment group and study period** | Number and proportion of patients with the first **non-surgical**  CD-related hospitalization | Number and proportion of patients with the first **surgical** CD-related hospitalization |
| --- | --- | --- |
| **Combined Induction, n (%)** |  |  |
| Overall | 52 (75.4) | 17 (24.6) |
| Risankizumab 600 mg IV | 11 (64.7) | 6 (35.3) |
| Risankizumab 1200 mg IV | 8 (80.0) | 2 (20.0) |
| Placebo IV | 33 (78.6) | 9 (21.4) |
| **Maintenance period, n (%)** |  |  |
| Overall | 16 (72.7) | 6 (27.3) |
| Risankizumab 180 mg SC | 5 (100.0) | 0 (0.0) |
| Risankizumab 360 mg SC | 7 (77.8) | 2 (22.2) |
| Placebo SC | 4 (50.0) | 4 (50.0) |

CD, Crohn’s disease; IV, intravenous; SC, subcutaneous.

**Appendix A: Study Designs of Induction and Maintenance Studies**

*Induction studies*

ADVANCE (NCT03105128) and MOTIVATE (NCT03104413) phase 3, double-blind, randomized clinical trials evaluated efficacy and safety of risankizumab as induction therapy. Eligible patients were aged ≥16 to ≤80 years with moderate-to-severe CD (Crohn’s Disease Activity Index [CDAI] 220–450, average daily soft stool frequency ≥4 and/or daily abdominal pain score ≥2, and endoscopic evidence of mucosal inflammation [Simple Endoscopic Score for CD [SES-CD] ≥6 [≥4 for isolated ileal disease] excluding the narrowing component]). Patients had inadequate response or intolerance to biologic therapy and/or conventional therapy in ADVANCE; in MOTIVATE, patients had inadequate response or intolerance to biologic therapy, with no more than 20% of patients having failed ustekinumab. Patients were permitted to receive concomitant corticosteroids if the dose was under a certain threshold (eg, prednisone <20 mg/day) and they were on the steroid course >14 days with a stable dose for at least 7 days; this stable dose was maintained throughout the induction period. Patients were randomized 2:2:1 in ADVANCE and 1:1:1 in MOTIVATE to receive intravenous (IV) risankizumab 600 mg, 1200 mg, or placebo. Patients received IV study drug at weeks 0, 4, and 8 during the 12-week induction period.^1^

*Maintenance study*

Briefly, the 52-week maintenance trial, FORTIFY (NCT03105102), was a phase 3, double-blind, re-randomized responder withdrawal study evaluating efficacy and safety of continuation of risankizumab as subcutaneous (SC) maintenance therapy relative to active treatment withdrawal in patients who responded clinically to 12-weeks IV risankizumab. Responders to IV risankizumab were re-randomized 1:1:1 to receive risankizumab 360 mg SC, 180 mg SC, or placebo SC (withdrawal from IV risankizumab) every 8 weeks for 52 weeks, from week 12 of induction (eg, week 0 of maintenance) onward. Tapering of concomitant corticosteroids was mandatory beginning at week 0, and initiation of or change in dosage (increase or decrease) of other concomitant CD medications was prohibited. For patients who met the criteria of increased symptoms and objective confirmation of disease activity, rescue therapy (open-label risankizumab 1200 mg IV for one dose followed by 360 mg SC every 8 weeks) was available.^2^

**Appendix B: Statistical Analysis**

Occurrence of CD-related hospitalizations after 12 weeks of induction treatment was assessed under overall type I error control at a significance level of α=0.05 (two-sided). As-observed occurrence of CD-related hospitalizations during the induction period was a binary endpoint (yes/no hospitalization occurred). Occurrences were reported as the number and proportion of patients with at least one CD-related hospitalization; 95% confidence intervals (CIs) for incidence rate were based on normal approximation to binomial distribution. *P-*values were based on a Chi-square test comparing proportions of patients with hospitalizations through 12 weeks between each risankizumab treatment group (ie, IV 600 mg or IV 1200 mg) and IV placebo.

Incidence rates for hospitalization during the maintenance period, among patients who were responders to risankizumab induction therapy, were calculated as number of patients with CD-related hospitalization events divided by time at risk (patient-years [PYs]) and are reported as incident rate per 100 PYs. Event date was defined as admission date for hospitalization. For patients with an event during the maintenance period, time at risk was defined as number of PYs from date of first maintenance dose of study drug post induction phase (ie, risankizumab 180 mg or 360 mg SC or treatment withdrawal placebo SC) to first hospitalization event before receipt of any risankizumab rescue therapy. For patients without a CD-related hospitalization event during the maintenance period or if rescue therapy was received before any event(s), time at risk was defined as number of PYs from first dose of study drug to the end of study follow-up period, where the follow-up period started from first dose date to date of risankizumab rescue visit if received rescue therapy, or date of last dose received during the maintenance period (+140 days if prematurely discontinued study drug or did not enter open-label extension period), or date of first dose of open-label extension period, whichever occurs earlier. Incidence rate difference, 95% Cis, and nominal *P*-value were calculated to evaluate statistical significance of the difference between each risankizumab SC groups and treatment withdrawal placebo SC group. Analysis was based on as-observed data before receiving risankizumab rescue therapy.

An integrated analysis was conducted to understand the effects of the complete risankizumab treatment regimen on reductions in hospitalization events. Primary integrated analysis incorporated exposure time and occurrence of CD-related hospitalization events in both induction and maintenance periods for labeled doses. Labeled doses included risankizumab 600 mg IV for induction and both 180 mg and 360 mg SC for maintenance in the United States. Patients were followed from the induction baseline in ADVANCE and MOTIVATE through the end of the maintenance period in FORTIFY with the same definition of time at risk described above. In addition, patients were censored if they did not achieve clinical response at week 12 or changed treatment from placebo to risankizumab during the induction period. The placebo group for the primary integrated analysis included all patients within the ITT 1A induction population who received IV placebo during the 12-week induction; these patients were censored at week 12. A sensitivity analysis was also conducted by accounting for exposure time and event for patients who were clinical responders to induction placebo IV and received placebo SC in the maintenance period. All analyses were performed using SAS Version 9.4 or later (SAS Institute Inc., Cary, NC).

**References**

1. D'Haens G, Panaccione R, Baert F, et al. Risankizumab as induction therapy for Crohn's disease: results from the phase 3 ADVANCE and MOTIVATE induction trials. *Lancet*. 2022;399:2015-2030.

2. Ferrante M, Panaccione R, Baert F, et al. Risankizumab as maintenance therapy for moderately to severely active Crohn's disease: results from the multicentre, randomised, double-blind, placebo-controlled, withdrawal phase 3 FORTIFY maintenance trial. *Lancet*. 2022;399:2031-2046.
